# Disclosure of Traditional and Complementary Medicine Use and Its Associated Factors to Medical Doctor in Primary Care Clinics in Kuching Division, Sarawak, Malaysia

**DOI:** 10.1155/2017/5146478

**Published:** 2017-04-26

**Authors:** Anak Kelak Johny, Whye Lian Cheah, Safii Razitasham

**Affiliations:** Department of Community Medicine & Public Health, Faculty of Medicine and Health Sciences, Universiti Malaysia Sarawak, Kuching, Sarawak, Malaysia

## Abstract

The decision by the patients to disclose traditional and complementary medicine (TCM) use to their doctor is an important area to be explored. This study aimed to determine the disclosure of TCM use and its associated factors to medical doctor among primary care clinic attendees in Kuching Division, Sarawak. It was a cross-sectional study using questionnaire, interviewer administered questionnaire. A total of 1130 patients were screened with 80.2% reporting using TCM. Logistic regression analysis revealed that being female (AOR = 3.219, 95% CI: 1.385, 7.481), perceived benefits that TCM can prevent complication of illness (AOR = 3.999, 95% CI: 1.850, 8.644) and that TCM is more gentle and safer (AOR = 4.537, 95% CI: 2.332, 8.828), perceived barriers of not having enough knowledge about TCM (AOR = 0.530, 95% CI: 0.309, 0.910), patient dissatisfaction towards healthcare providers being too business-like and impersonal (AOR = 0.365, 95% CI: 0.199, 0.669) and paying more for healthcare than one can afford (AOR = 0.413, 95% CI: 0.250, 0.680), and accessibility of doctors (AOR = 3.971, 95% CI: 2.245, 7.023) are the predictors of disclosure of TCM use. An open communication between patients and doctor is important to ensure safe implementation and integration of both TCM and medical treatment.

## 1. Introduction

The use of traditional and complementary medicine (TCM), a group of diverse medical and healthcare practices and products that are not considered part of conventional medicine, has increased in popularity within the world including Malaysia in the past decade [1–3]. Estimate of TCM use by Malaysian's adults range from 67.6% to 71.2% in their lifetime and 53.8% to 57.4% within the last period of 12 months of the study [3]. Traditional and complementary use globally including Malaysia is expected to continue to grow as global shifts occur in the focus on preventive medicine and individual control over healthcare options [1, 4].

Demand for TCM products, practices, and practitioners worldwide is substantially increasing and Malaysia is no exception. According to Health Informatics Centre, Ministry of Health Malaysia, there were approximately 11,691 TCM practitioners all over Malaysia and this number approximately increased to 15,000 in the year 2011 [5]. Furthermore, TCM Division received RM 2,185,460.00 (US$ 520348) budget which is much higher than in 2006 and 2007 for operational and development expenditure and 90.75% of the budget was spent on management services such as payment to traditional and complementary medicine practitioners and acquisition of supplies for TCM integrated hospitals [6]. Moreover, out of pocket TCM expenditure is rapidly growing. It is estimated that Malaysia spends approximately US$500 million annually on traditional therapies, compared to only US$ 300 million on conventional therapies [7]. This increasing utilization of TCM especially in Malaysia may lead to a situation in which people are not only using TCM, but also substituting conventional medicine with TCM suggesting that it is important to understand why people choose to use TCM.

Despite the increasing popularity of TCM use, very little consensus has been reached regarding the decision to disclose the use of TCM among TCM users to their conventional healthcare provider [8–10]. While many traditional and complementary medicine therapies have great health benefits, many may have negative side effects or even interfere with conventional medical practices. As a whole, individuals who use TCM are not likely to discuss their use of these practices with their medical providers and therefore placing themselves at risk of undue harm [7]. Due to the possible effects of interactivity that may lead to inaccurate biochemical assay effect, the physicians may increase the drug dosage to dangerous levels or reduce them to levels at which the drugs are ineffective [11]. Such concerns are plausible because people with chronic illness have been found to often use TCM therapies concurrently with biomedicine [12]. Consequently, understanding the behavior of people who use TCM and biomedicine concurrently is particularly pertinent because nondisclosure of TCM therapies to conventional healthcare professionals is noted to be as high as 66% to 70% [13].

Moreover, there is very little information about the variables associated with TCM disclosure possibly because the main focus of TCM research has been on its use. What little information is known about TCM disclosure has come from studies on TCM use, which suggested the patient-doctor encounter and sociodemographics were associated with TCM disclosure [4, 14]. Furthermore, most studies have minimally addressed the communication experiences that underlie such reasoning and therefore do not substantially explain the process by which participants select to disclose TCM [15]. To better understand these factors it is necessary to consider the influences that shape and drive healthcare decisions which to date have not been explored as experience of disclosure of TCM use. In addition, local folk remedies can pose substantial culture-centric sensitivity among patients who attended primary health centers in disclosing TCM use, particularly in Sarawak that has a multiethnicity composition. We addressed the following research question; what is the rate of disclosure of TCM use and factors associated with the decision for disclosure of TCM use to medical doctor among primary care clinic attendees in Kuching Division, Sarawak? Thus, this study aimed to determine the disclosure of TCM use and its associated factors to medical doctor among primary care clinic attendees in Kuching Division, Sarawak.

## 2. Materials and Methods

### 2.1. Study Design and Sample

It was a cross-sectional study conducted in Kuching Division, Sarawak, Malaysia, using interviewer administered questionnaire. The interview was undertaken between August and October 2016 in the randomly selected primary healthcare clinic in Kuching Division, Sarawak. Sarawak is the largest state in Malaysia, with a population of approximately 2.8 million, and it is the least densely populated state among 13 states in Malaysia. Kuching Division is one of the eleven administrative divisions in Sarawak and it consists of three administrative districts, Kuching, Bau, and Lundu, and three subdistricts, Padawan, Siburan, and Sematan.

Healthcare in Sarawak is administratively under Sarawak Health Department, delivered through a network of static and mobile facilities that provide promotive, preventive, curative, and rehabilitative care. In Kuching Division, there are 22 health clinics, 9 maternal and child health clinics, 3* Klinik Desa *(community clinic), 4 mobile health teams, 1 Flying Doctor Service, 3 1 Malaysia Clinics, and 111 Village Health Promoters [16]. In line with the global focus on TCM, Sarawak General Hospital is one of the integrated hospitals for practicing TCM since 2010 [17]. To date, TCM in Sarawak General Hospital only provides acupuncture, Malay massage, and postnatal massage services.

The inclusion criteria for selecting respondents were Malaysian citizen aged 18 years and above, registered patients at the outpatient government primary health clinics that have medical doctors, using at least one of TCM based on classification of TCM in Malaysia [18] in the past 12 months or lifetimes, and ability to understand either in English or in Malay language.

Using OpenEpi (Open Source Epidemiologic Statistics for Public Health) (OpenEpi, 2015), based on sampling frame of 671,802 (2010 census of total population of Kuching Division) [19] and prevalence of disclosure of TCM of 43% [14], a total of 906 respondents were needed for this study (attrition of 20%). From there, each government primary health clinic in three districts was proportionated based on yearly census health clinic attendances for the selection of total sample unit (respondent). Within every government health clinic, universal sampling on every traditional and complementary medicine user in outpatient department on Monday until Friday was selected until it reaches minimum sample size.

### 2.2. Survey Instrument

The data and information from the respondent was determined using an interviewer administered questionnaire. The questionnaire was developed based on “Health Belief Model” and “Anderson's Health Behavioral Model” that involved 7 sections: (1) type of TCM use and disclosure of TCM use; (2) sociodemographic and economic characteristics; (3) health profile of respondents; (4) healthcare utilization; (5) attitude towards traditional and complementary medicine use; (6) healthcare general satisfaction level; and (7) patient-doctor relationship status. In the first section, respondents were first asked about their use of TCM: “Have you ever used any traditional and complementary medicine for your own health or treatment as well as in the previous 12 months?” The checklist of 6 specific TCM modalities based on the classification of TCM modalities by TCM Division, Ministry of Health Malaysia [18], including traditional Malay medicine, traditional Chinese medicine, traditional Indian medicine, manipulative based practice, energy medicine, mind, body, and soul therapy, and biological based medicine, was used to stimulate respondents' memory during the interviews. Respondents who replied “no” to that question were excluded from this study. The disclosure of those respondents who answer “yes” was ascertained using the question: “Did you let any medical doctor know about your use of TCM?” Thus, disclosure of TCM to their medical doctor was determined as the dependent variable of this study. In the second section, the sociodemographic and economic characteristics were collected in relation to age, gender, marital status, ethnicity, religions, level of education, employment status, income, and insurance coverage. In the third section, health profile of respondents, data were collected on chronic medical condition and self-rated health status. The self-rated health status of respondents was assessed by asking them: “In general, how would you rate your health today?” with the possible choices being “ very good” (1), “good” (2), “moderate” (3), “bad” (4), or “very bad” (5). In the fourth section, data on healthcare utilization was collected based on the number of clinic visits and hospitalization for the past 12 months. In the fifth section, to assess the attitude towards TCM, 16 items on a 5-point Likert scale instrument from (1) strongly disagree to (5) strongly agree on perceived benefits with a range score from 24 to 54 and barrier with a range score from 8 to 24 were adopted from Chang et al. [20]. In the sixth section, for patient satisfaction towards healthcare service component, the validated Short-Form Patient Satisfaction Questionnaire (PSQ-18) was adopted from Ganasegeran et al. [21]. The PSQ-18 is comprised of 7 domains including (1) general satisfaction; (2) technical quality; (3) interpersonal manner; (4) communication; (5) financial aspect; (6) time spent with doctor, and (7) accessibility and convenience. These items were scored on a 5-point Likert scale ranging from (1) strongly agree to (5) strongly disagree with the interval score of patient satisfaction score of all respondents being from 14.0 to 31.5. In the seventh section, Patient-Doctor Relationship Questionnaire-9 (PDRQ-9), a 9-item questionnaire adopted from Van Der Feltz-Cornelis et al. [22] was used. The appropriateness of the statements was simplified to a 5-point Likert scale of (1) not at all appropriate to (5) totally appropriate with the interval score of patient-doctor relationship score of all respondents being from 18 to 45.

The developed English questionnaire was translated into Malay language using backward-translation method and validated by two bilingual professionals. Both individuals are the academicians in local learning institutes in Sarawak who are fluent in both Malay and English. At this stage, any items that appeared discrepant to the meaning of the original items were translated again. After the questionnaire was drafted, the questionnaire was prepared in dual language (i.e., English and Malay language) and pilot study was conducted using the final questionnaire. A pilot testing of the survey was conducted interviewing 40 eligible respondents in the primary healthcare clinic* (Klinik Kesihatan Kota Samarahan)* in Samarahan Division, Sarawak. These samples were not included in the actual study. The content validity of the survey instrument was established by a panel of experts. Two academic professionals and biostatisticians were expert in TCM, and research method evaluated each survey item and only a few items, which had low Cronbach's alpha coefficient, were revised or rephrased accordingly. Overall, the pilot study results showed Cronbach's alpha coefficient ranging from 0.617 to 0.948, indicating the items have adequate internal consistency and were reliable.

### 2.3. Data Collection Procedures

The data was collected by using interviewer administered questionnaire. The interview was conducted in primary healthcare clinic in Kuching Division, Sarawak. Information on the patient was provided by the clerk in-charge at the registration counter to the researcher. Patients were approached while waiting for consultation with the doctors in the waiting room. After determining that potential respondents met the inclusion criteria, the information sheet and the consent form were discussed and signed. They had been informed that they could withdraw from the study at any time, and this would not affect the healthcare services they receive in any way. During the data collection, all the questionnaires were answered either in Malay or in English according to their preferences. The interview was taken in approximately 15 to 20 minutes for each respondent.

### 2.4. Statistical Analysis

The survey data was checked, verified, entered, and analyzed using Statistical Package for Social Sciences (SPSS) version 20. Descriptive and inferential statistics was carried out to answer the research objectives. The findings were described in terms of frequencies, percentages, means, and standard deviations. Bivariate relationship was performed to determine the association between the potential predictors and dependent variables (disclosure of TCM use). Chi-square test was used for qualitative variables and Student's *t*-test was used to analyze quantitative and qualitative variables. In order to determine predictors of disclosure of TCM use, Multiple Logistic Regression Analysis was employed. The level of statistical significant was indicated by a *p* value of 0.05 or less and two-tailed test was used for all of the statistical analysis.

### 2.5. Ethical Approval

The ethical approval was obtained from the Ethics Committees from the Universiti Malaysia Sarawak (UNIMAS) and National Medical Research Register (NMRR) Ministry of Health Malaysia (NMMR-16-178-29371). A written consent was obtained from those who agreed to participate in this study and their anonymity and confidentiality were assured.

## 3. Results

A total of 1130 patients were screened for TCM use and 906 patients (80.2%) reported using TCM. Out of this, all 906 respondents have at least 1 type of TCM modalities in the past 12 months or lifetime and agreed to participate, giving a response rate of 100%.

### 3.1. Characteristics of Sample

Characteristics of the sample including sociodemographic and economic information are presented in Table 1. The majority of the respondents were from age group of 41–60 years old, with more males and Malays. More than 45% of the respondents were unemployed and majority were from monthly income group of ≤RM1000 (69.6%).

### 3.2. Health Profile of the Respondents

Information on the health profile of the respondents is presented in Table 2. Majority of the participants (95.5%) had at least 1 medically diagnosed chronic disease. The three most common diseases were hypertension (85.1%), followed by diabetes mellitus (62.7%) and hypercholesterolemia (50.6%). Out of these, more than one-third (42.4%) of the respondents had a combination of 3 of these chronic diseases. More than two-thirds (79.1%) of the participant rated their health as poor to fair. The mean frequency of clinic visits within 12 months was 2.8 (0.6) times.

### 3.3. TCM Use Profile and Disclosure of TCM Use

Table 3 shows the TCM use profile and disclosure of TCM use of the respondents. Majority of the respondents (97.3%) use at least 1 type of TCM in their lifetime. The commonest type of TCM use within 12 months was biological based medicine (mostly on nutritional therapy) (43.60%), followed by traditional Malay medicine (18.43%) (mostly Malays herbs),* Urut Melayu *(Malay massage), or* Bekam* (cupping), and traditional Chinese medicine (5.96%) (mostly on Chinese herbs, acupuncture, and Tai Chi). Only 9.6% of the respondents claimed that they had disclosed either the use of TCM in their lifetime or current use within previous 12 months.

### 3.4. Attitude towards TCM Use, Patients Satisfaction, and Patient-Doctor Relationship Status of the Respondents

Table 4 presents the perceived benefits, perceived barriers, patient satisfaction, and patient-doctor relationship according to the respondents. Respondents answered a total of 11 questions on perceived benefits towards TCM use which had a total score of 55; the mean score of perceived benefits towards TCM use for all respondents was 38.8 (4.37). Meanwhile, respondents answered a total of 5 questions on perceived barriers towards TCM use which had a total score of 30; the mean score of perceived barriers towards TCM use for all respondents was 16.60 (2.82). For patient satisfaction level, respondent answered a total of 18 questions, which had a total score of 35; the mean score of PSQ-18 for all respondents was 21.74 (3.33). Meanwhile, respondents answered a total of 9 questions on patient-doctor relationship, which had a total score of 45; the mean score of PDRQ-9 for all respondents was 30.60 (5.99).

### 3.5. Relationship between Disclosure of TCM Use with the Sociodemographic and Economic Characteristics, Attitude towards TCM Use, Patient Satisfaction Level, and Patient-Doctor Relationship Status of the Respondents

In order to determine the relationship among the potential predictors (independent variables) and dependent variable, bivariate analysis was performed by using independent *t*-test and Chi-squared test of independence. Preliminary analysis was conducted to ensure no violation of assumption of normality, linearity, and homoscedasticity. Outliers were then identified and removed. Thus, the total number of cases which remained for further analysis was 862.  Table 5 shows that there were only significant differences between the respondent's age in years, gender, number of TCM modalities used, attitude towards TCM use score, patient satisfaction score, patient-doctor relationship score, and disclosure of TCM use.

### 3.6. Predictors of Disclosure of TCM Use of the Respondents

For the logistics regression analysis, seven (7) independent variables were identified as important predictors in the final full explainable model: gender; perceived benefits towards TCM use (item-5 and item-15); perceived barriers towards TCM use (item-9); patient satisfaction level (item-9 and item-13); and patient-doctor relationship status (item-9) (Table 6).

The model containing the seven (7) independent variables explained 24.0% (Cox and Snell *R* square) and 58.8% (Nagelkerke *R* square) of the variance in the disclosure of TCM use. It was also able to classify 95.4% of the cases. The goodness of fit indices were not statistically significant (*p* > 0.05) which indicated that the assumption was not violated.

Table 6 presents that being female (AOR = 3.219, 95% CI: 1.385, 7.481), perceived benefits that TCM can prevent complication of illness (AOR = 3.999, 95% CI: 1.850, 8.644), perceived benefits that TCM is more gentle and safer (AOR = 4.537, 95% CI: 2.332, 8.828), perceived barriers of not having enough knowledge to select the right TCM (AOR = 0.530, 95% CI: 0.309, 0.910), patient dissatisfaction towards healthcare providers because they act too business-like and impersonal (AOR = 0.365, 95% CI: 0.199, 0.669), patient dissatisfaction towards paying more for healthcare than one can afford (AOR = 0.413, 95% CI: 0.250, 0.680), and accessibility of doctors (AOR = 3.971, 95% CI: 2.245, 7.023) appeared to be important predictors factors of disclosure of TCM use (*p* < 0.05).

Therefore, this study showed that the disclosure of TCM use was 3.2 times likely higher among female participants than male. In terms of attitude towards TCM use, for any increased score for the perceived benefits that TCM prevents complication of illness and the use of TCM is more gentle and safer, the odds of him/her reporting a disclosure of TCM use to medical doctor increase by a factor of 4.0 and 4.5, respectively. However, for any increased score for the perceived barriers of TCM of having no knowledge about TCM, the odds of him/her reporting a disclosure of TCM use to medical doctor decrease by a factor of 0.5.

In terms of patient satisfaction level, for any increased score for doctor's manner (acting too business-like and impersonal) and paying more for healthcare, the odds of him/her reporting a disclosure of TCM use to medical doctor decrease by a factor of 2.7 and 2.4, respectively. Meanwhile, in terms of patient-doctor relationship, for any increased score for accessibility of doctors, the odds of him/her reporting a disclosure of TCM use to medical doctor increase by a factor of 4.0.

## 4. Discussion

In this study, it was found that the rate of disclosure of TCM use to medical doctor among primary care clinic attendees in Kuching Division, Sarawak, was 9.6%. This rate was found to be consistent with another study by Sleath et al. [23] where 10% of their participants had discussed alternative therapies with doctors. However, in a study carried out in Taiwan, only 2.0% of the TCM users disclosed their TCM use to their doctor [24]. Existing literature suggested that there are inconsistence findings between TCM use and its disclosure to the doctors [8–10]. Such pattern is not uncommon because the patients may not have adequate knowledge about which type of TCM is legitimate and acceptable to doctors. This is evidenced in the type of TCM use in this study where 43.6% of the respondents had used biological based medicine such as nutritional therapy and naturopathy. Patients may be unaware of the possible interaction between drugs and biological based medicine and may consider it is unimportant to inform their doctors. Moreover, it is not a common practice that the doctor asks patients about their use of TCM. Language and cultural barriers between doctor and patients may be another possible reason that could create communication breakdown and thus reducing the rate of disclosure [25]. In this study, although the common national language used is Malay language, not all patients can communicate well particularly since the proportion of non-Malay was 44.5% (Chinese, Bidayuh, and others).

Female was found 3.219 times more likely to disclose TCM usage to doctor than male, consistent with previous studies [15, 26, 27]. It was suggested that compared to males, females are more subjected to medical encounter and have better treatment compliance, which explains why they have more opportunity to discuss their health with doctor [28]. This can be reflected in the higher number of female respondents in this study compared to male respondents (65.9% versus 34.1%).

In terms of attitude towards TCM use, the respondents believed that using TCM can prevent their illness complication, and it is gentle and safer to use. Such practices are not uncommon; especially majority of the respondents in this study were diagnosed to have at least one type of chronic disease such as hypertension (85.1%), diabetes mellitus (62.7%), and hypercholesterolemia (50.6%). Using biological based medicine such as nutritional therapy and naturopathy, the respondents believed that it can boost and maintain their health status. This situation is common to other chronic diseases such as cancer patients where vitamin and health supplements were used to reduce cancer symptoms and unwanted side effects of cancer treatment [29, 30].

Lower perception of barriers of no knowledge on selection of the right TCM was another predictor of disclosure of TCM use. Patients are reluctant to disclose their use of TCM because they are afraid that whatever they consume might interfere with their treatment. It is important to note that most of the TCM are not prescribed by medical certified personnel. The main source of TCM use is often through the advice and recommendation of friends and family members [31]. Ministry of Health Malaysia in recent years had carried out some enforcement activities through the Traditional and Complementary Medicine Division and reported several false claims on some of the TCM products in Malaysia [17].

The findings of this study indicated that patients who are dissatisfied with healthcare providers who act too business-like and impersonal and dissatisfied with paying for more healthcare than one can afford are more likely to have better patient-doctor relationship, which will increase the rate of disclosure of TCM use to their doctor. Particularly in this case, the majority of the respondents were from monthly income group of RM1000 and below. Nevertheless, it is important to note that the healthcare service in all study sites and any government healthcare centers are subsidized by the Malaysia Government.

In this study, patient opinions on accessibility of doctor can predict the disclosure of TCM use. This can be reflected in the higher number of clinic visits of more than 3 times (84.7%) a year as all the government healthcare centers in these study sites have at least one medical doctor in-charge. Patient who decided to disclose TCM use believed their doctors are more knowledgeable on TCM and appear to put trust on their doctors regarding the safe use of TCM [14]. Hence, effective physician-patient relationship and discussions on TCM use are crucial in order to reduce the dangers of TCM-drug interactions [32].

While the current study has contributed to a better understanding of the predictors of TCM use among primary care clinic attendees in Kuching Division, Sarawak, Malaysia, it has several limitations. The nature of cross-sectional study cannot determine the causal relationship between predictors and outcomes. It also does not reflect the changing process of decision-making of TCM use, such as disclosure of TCM over time. The study was conducted in Kuching Division, Sarawak; therefore it does not represent the general population of disclosure of TCM use in Sarawak or Malaysia.

## 5. Conclusion

In conclusion, the study had indicated some of the determining factors influencing the disclosure of TCM use among primary care clinic patients in Kuching Division, Sarawak, that can help to further strengthen the management of healthcare particularly in the front line when the interaction between doctor and patients is involved. It also underscores the need for healthcare providers to regularly screen and perform further assessment of the frequency and the use of TCM. An increased awareness of the importance of these issues assists healthcare professionals to provide more appropriate and effective care, including culturally sensitive and supportive environment during consultations. In addition, an open communication between patients and medical doctor in regard to the use of TCM is an important key to ensuring safe implementation and integration of both TCM and medical treatment.

## Figures and Tables

**Table 1:** Sociodemographic and economic characteristic of the participants (*N* = 906).

Characteristics	*n* (%)	Mean (SD)
*Age in years*		47.91 (9.14)
≤20	2 (0.2)	
21–30	35 (3.9)	
31–40	151 (16.7)	
41–50	351 (38.7)	
51–60	298 (32.9)	
>60	69 (7.6)	
*Gender*		
Female	597 (65.9)	
Male	309 (34.1)	
*Ethnicity*		
Malay	503 (55.5)	
Chinese	228 (25.2)	
Bidayuh	154 (17.0)	
Others	21 (2.3)	
*Religions*		
Muslim	504 (55.6)	
Christian	319 (35.2)	
Buddhism	82 (9.1)	
No religion	1 (0.1)	
*Education*		
No formal education	65 (7.2)	
Primary	628 (69.3)	
Secondary	189 (20.9)	
Tertiary	24 (2.6)	
*Marital status*		
Married (living with partner)	833 (91.9)	
Single (never been married)	55 (6.1)	
Widowed	18 (2.0)	
*Occupations*		
Unemployed	420 (46.4)	
Own business	375 (41.4)	
Private employee	86 (9.5)	
Government employee	25 (2.8)	
*Household income per month (Ringgit Malaysia RM)*		1087.22 (837.35)
≤RM1000	631 (69.6)	
RM1001–RM2000	229 (23.3)	
RM2001–RM3000	28 (3.1)	
RM3001–RM4000	6 (0.7)	
RM4001–RM5000	8 (0.9)	
>RM5000	4 (0.4)	
*Health insurance coverage*	33 (3.6)	

**Table 2:** Health profile and healthcare utilization of the participants (*N* = 906).

	*n* (%)	Mean (SD)
*Presence of chronic disease* (*n* = 906)	865 (95.5)	
*Type of chronic diseases* (*n* = 865)^*∗*^		
Hypertension	771 (85.1)	
Diabetes mellitus	568 (62.7)	
Hypercholesterolemia	458 (50.6)	
Asthma	62 (6.8)	
Arthritis	24 (2.6)	
Heart disease	5 (0.6)	
Stroke	8 (0.9)	
Cancer	1 (0.1)	
Others	3 (0.3)	
*Numbers of chronic diseases *		
0	41 (4.5)	
1	273 (30.1)	
2	177 (19.5)	
3	384 (42.4)	
4	31 (3.4)	
*Self-rated health status*		
Poor-fair	717 (79.1)	
Good-excellent	189 (20.9)	
*Frequency of clinic visits within 12 months*		2.80 (0.63)
*Hospitalization within 12 months*	69 (7.6)	
*Frequency of hospitalization within 12 months (1–3 times)*	69 (7.6)	

^*∗*^Multiple responses.

**Table 3:** TCM use and disclosure of TCM use of the respondents (*N* = 906).

	*n* (%)
*Number of TCM modalities used (ever used) 1–3 types*	881 (97.3)
*TCM modalities used within 12 months* ^*∗*^	
* Biological based medicine* (nutritional therapy, naturopathy)	395 (43.6)
* Traditional Malay medicine *(Malay herbs, *Urut Melayu*, and *Bekam*)	167 (18.4)
* Traditional Chinese medicine* (Chinese herbs, acupuncture, Tai Chi, and Qi Gong)	54 (6.0)
* Manipulative based practice* (chiropractor, osteopathy, massage, spa therapy, reflexology, and aromatherapy)	41 (4.5)
* Traditional Indian medicine* (Ayurveda, Siddha, Unani, and Yoga/meditation)	2 (0.2)
*Disclosure of TCM use*	87 (9.6)

^*∗*^Multiple responses.

**Table 4:** Attitude towards TCM use, patients satisfaction, and patient-doctor relationship status of the respondents (*N* = 906).

	Mean	SD	Min–max score
*Attitude towards TCM use*			
Perceived benefits	38.8	4.37	24–54
Perceived barriers	16.6	2.82	8–24
*Patient satisfaction score*	21.74	3.33	14.0–31.5
*Patient-doctor relationship score*	30.60	5.99	18–45

**Table 5:** Relationship between disclosure of TCM use with the sociodemographic and economic characteristics, attitude towards TCM use, patient satisfaction level, and patient-doctor relationship status of the respondents (*N* = 862).

Characteristics	*n*	Disclosure of TCM use		*p* value
		Yes (*n* = 63)	No (*n* = 799)	
*Sociodemographic and economic characteristics*				
Age in years				
Mean (SD) years		45.92 (5.29)	48.28 (8.89)	^ii^0.002^*∗∗*^
Gender				
Female	571	8.8	91.2	^i^0.022^*∗*^
Male	291	4.5	95.5	
Ethnicity				
Malay	479	5.4	94.6	^i^0.117
Chinese	216	9.3	90.7	
Bidayuh	151	9.9	90.1	
Others	16	12.5	87.5	
Religions				
Muslim	479	5.4	94.6	^i^0.123
Christian	302	9.6	90.4	
Buddhism	80	10.0	90.0	
No religion	1	0.0	100.0	
Education				
No formal education	64	7.8	92.2	^i^0.553
Primary	613	7.0	93.0	
Secondary	168	8.9	91.1	
Tertiary	17	0.0	100.0	
Marital status				
Married (living with partner)	796	7.3	92.7	^i^0.378
Single (never been married)	49	10.2	89.8	
Widowed	17	0.0	100.0	
Occupation				
Unemployed	407	7.9	92.1	^i^0.242
Own business	356	6.2	93.8	
Private employee	80	11.3	88.8	
Government employee	19	0.0	100.0	
Household income per months				
Mean (SD) RM		1096.83 (811.01)	1065.92 (811.37)	^ii^>0.05
Health insurance coverage				
Yes	31	6.5	93.5	^i^0.852
No	831	7.3	92.7	
*Health profile*				
Presence of chronic diseases				
Yes	826	7.5	92.5	^i^0.286
No	36	2.8	97.2	
Number of chronic diseases				
Mean (SD)		2.27 (0.94)	2.11 (1.01)	^ii^>0.05
Self-rated health status				
Poor-fair	694	8.1	91.9	^i^0.081
Good-excellent	168	4.2	95.8	
*Healthcare utilization*				
Frequency of clinic visits within 12 months				
Mean (SD) times		2.87 (0.52)	2.81 (0.61)	^ii^>0.05
Hospitalization within 12 months				
Yes	66	7.6	92.4	^i^0.931
No	796	7.3	92.7	
Frequency of hospitalization within 12 months				
Mean (SD) times		0.08 (0.27)	0.08 (0.31)	^ii^>0.05
Number of TCM modalities used (ever used)				
Mean (SD) types		1.30 (0.61)	1.55 (0.79)	^ii^0.004^*∗*^
*Attitude towards TCM use*				
Perceived benefits		44.06 (3.27)	38.31 (3.73)	^ii^<0.001^*∗∗∗*^
Mean (SD) score				
Perceived barriers		14.59 (1.86)	16.90 (2.75)	^ii^<0.001^*∗∗∗*^
Mean (SD) score				
*Patient satisfaction level*				
Mean (SD) score		25.37 (1.55)	21.30 (3.19)	^ii^<**0.00**1^*∗∗∗*^
*Patient-doctor relationship status*				
Mean (SD) score		37.75 (3.19)	29.92 (5.71)	^ii^<0.001^*∗∗∗*^

^*∗*^ *p* < 0.05; ^*∗∗*^*p* < 0.01; ^*∗∗∗*^*p* < 0.001.

^i^ *p* value reached from Chi-square test.

^ii^ *p* value reached from independent *t*-test.

**Table 6:** Factor affecting the disclosure of TCM use (*N* = 862).

Independent variables	*β*	SE	Adjusted odds ratio	95% CI	
				Lower limit	Upper limit
*Gender*					
Male (RC)			**1**		
Female	1.169^*∗*^	0.430	**3.219**	1.385	7.481
^a^ *Attitude towards TCM use*					
Perceived benefits					
Preventing illness complications	1.386^*∗∗*^	0.393	**3.999**	1.850	8.644
Treatment is more gentle and safer	1.512^*∗∗*^	0.340	**4.537**	2.332	8.828
Perceived barriers					
Not having enough knowledge to select the right TCM	−0.634^*∗*^	0.275	**0.530**	0.309	0.910
^b^ *Patient satisfaction level*					
Healthcare providers act too business-like and impersonal	−1.009^*∗*^	0.309	**0.365**	1.496	5.027
Paying more for healthcare than one can afford	−0.885^*∗*^	0.255	**0.413**	1.471	3.992
^c^ *Patient*-*doctor relationship status*					
I find my doctor easily accessible	1.379^*∗∗*^	0.291	**3.971**	2.245	7.023

*Constant *	−23.809^*∗∗*^	**2.778**	**0.000**		

Model Chi-square (*df*)	236.016^*∗∗*^ (**7**)				
*n*	**862**				
Hosmer and Lemeshow Test	*p* > 0.05				

^*∗*^ *p* < 0.05; ^*∗∗*^*p* < 0.001.

Hosmer and Lemeshow Test, *X*^2^(*dF*) = 6.738 (8); *p* = 0.565.

Cox & Snell *R* square = 0.240. Nagelkerke *R* square = 0.588.

RC = reference category.

Dependent variable = disclosure of TCM use (yes versus no).

Forward logistic regression method was used for analysis.

## Abbreviations

TCM: Traditional and complementary medicine
SPSS: Statistics Package for Social Science
PSQ-18: Patient Satisfaction Questionnaire-18
PDRQ-9: Patient-Doctor Relationship Questionnaire-9.
